# TNFα and IL-17 cooperatively stimulate glucose metabolism and growth factor production in human colorectal cancer cells

**DOI:** 10.1186/1476-4598-12-78

**Published:** 2013-07-17

**Authors:** Daniel S Straus

**Affiliations:** 1 Biomedical Sciences Division, School of Medicine, University of California, Riverside, CA 92521, USA

**Keywords:** Colorectal cancer, TNFα, IL-17, HIF-1α, Aerobic glycolysis, NF-κB, SLC2A1, Hexokinase-2

## Abstract

**Background:**

Inflammation is a well-known etiological factor for colorectal cancer, but mechanisms underlying the linkage between inflammation and cancer are incompletely understood. We hypothesized that two pro-inflammatory cytokines, TNFα and IL-17, might play a role in promoting colorectal carcinogenesis. Aerobic glycolysis is a metabolic adaptation that promotes the survival/proliferation of cancer cells. Paracrine signaling between tumor cells and cancer-associated fibroblasts also plays a role in carcinogenesis.

**Methods:**

The effect of TNFα and IL-17 on aerobic glycolysis and growth factor production in cultured human colorectal cancer cells was investigated. Glucose utilization and lactate production were quantified by measuring the disappearance of glucose and appearance of lactate in the culture medium. Glucose transporter and glycolytic enzyme expression levels were measured by immunoblotting.

**Results:**

TNFα and IL-17 cooperatively stimulated glycolysis in HT-29, T84, Caco-2 and HCT116 colorectal cancer cells. Treatment of HT-29 cells with TNFα plus IL-17 also increased the expression of HIF-1α and c-myc, two factors know to induce the transcription of genes encoding components of the glycolytic pathway. To further investigate mechanisms for cytokine-stimulated glycolysis, the effects of TNFα and IL-17 on expression of six members and one regulator of the glycolytic pathway were investigated. TNFα and IL-17 cooperatively increased the expression of the glucose transporter SLC2A1 and hexokinase-2 but did not regulate expression of glucose transporter SLC2A3, enolase-1, pyruvate kinase M2, lactate dehydrogenase A, or 6-phoshofructo-2-kinase/fructose-2,6-bisphosphatase-3 (PFKFB3). Experiments with inhibitors indicated that HIF-1α played a role in induction of SLC2A1 and that the transcription factor NF-κB played a role in induction of hexokinase-2 by TNFα and IL-17. TNFα and IL-17 also synergistically stimulated production by HT-29 cells of a growth factor that simulated proliferation/survival of NIL8 fibroblastic cells. The activity of this factor was not specifically inhibited by the EGFR inhibitor AG1478, indicating that it is not an EGFR ligand.

**Conclusions:**

Chronic inflammation is known to promote colorectal tumorigenesis. The pro-inflammatory cytokines TNFα and IL-17 may contribute to this effect by stimulating glycolysis and growth factor production in colorectal cancer cells.

## Introduction

Considerable evidence has accumulated recently implicating inflammation as a causative factor in tumorigenesis [1,2]. There were an estimated 142,570 new cases of colorectal cancer (CRC) in the US in 2010, and CRC is the second most common cause of cancer death in the US [3]. A role for inflammation in causation of CRC is well documented [4,5]. For example, patients with inflammatory bowel disease (ulcerative colitis and Crohn’s disease) have a greatly increased risk for colorectal cancer [4,5]. Moreover, non-steroidal anti-inflammatory drugs such as aspirin and cyclooxygenase inhibitors have been shown to decrease the occurrence of adenomatous polyps [6]. However, despite clear evidence implicating inflammation in causation of CRC and other cancers, molecular mechanisms underlying this phenomenon are incompletely understood.

Much interest has focused recently on metabolic abnormalities in cancer cells. Among these, aerobic glycolysis (i.e. the Warburg effect) is a metabolic adaptation that promotes the survival/proliferation of cancer cells [7-9]. Increased activity of the glycolytic pathway provides biosynthetic substrates required by proliferating cells, inhibits apoptosis, and results in increased production of L-lactate, which exerts pro-carcinogenic effects [7-10]. While increased expression/activity of transcription factors HIF-1α and c-myc is thought to play an important role in the increased glycolysis in cancer cells, the underlying mechanisms are not completely understood [11-13]. HIF-1α is a master regulator of genes encoding components of the glycolytic pathway [11,12], and c-myc also positively regulates some of these same genes [13-15]. High activity of the PI3 kinase-AKT signaling pathway in cancer cells appears to be causally related to the increased expression of HIF-1α and c-myc [13]. Growth factors such as EGF and insulin increase the expression of HIF-1α [16,17], and a few studies have implicated cytokines as regulators of HIF-1α [18-20].

The two cytokines, TNFα and interleukin 17 (IL-17, also called IL-17A), play an important role in both acute and chronic inflammation. The importance of TNFα in inflammatory bowel disease is illustrated by the efficacy of anti-TNFα monoclonal antibodies in treating Crohn’s disease and ulcerative colitis [21]. In the gut, IL-17 is produced by a number of innate immune cells including innate lymphoid cells (ILC) [22,23]. In the adaptive immune response, IL-17 is produced by Th17 cells [24]. IL-17 expression is increased in inflammatory bowel disease [25], and tumor-infiltrating Th17 cells are found in human colorectal cancer and are associated with shortened disease-free survival [26,27]. Recent studies with mouse models have also revealed a role for IL-17 signaling in the development of colorectal tumors [28]. TNFα is produced in colorectal tumors by infiltrating macrophages, and higher TNFα expression is correlated with increased tumor diameter [29]. Thus, TNFα and IL-17 are frequently both present in acute and chronic inflammation, and both have been linked to colorectal cancer. The combined effect of the two cytokines on tumor cell metabolism and growth is therefore of considerable interest.

Previous studies of the regulation of gene expression by TNFα plus IL-17 have shown cooperative effects, in which TNFα induces transcription of target genes while IL-17 stabilizes their mRNAs [30,31]. The transcription factor NF-κB is an important mediator of transcriptional effects of TNFα in target cells including colorectal cancer cells [30]. Moreover, IL-17 synergizes with TNFα to induce expression of a number of genes including those encoding chemokines such as CXCL1, CXCL8, and CCL20 [30]. Major mechanisms for this effect are activation of the canonical NF-κB signaling pathway by TNFα resulting in increased gene transcription, and IL-17-mediated stabilization of mRNAs [30,31].

Heterotypic interactions between tumor cells and stromal cells in the surrounding microenvironment play an essential role in tumorigenesis [32]. Stromal cancer-associated fibroblasts (CAF) and tumor cells form a reciprocal positive feedback loop, in which the tumor cells produce factors that promote activation, proliferation and chemotaxis of CAF, which in turn produce factors that enhance tumor cell proliferation [33-35]. Inhibition of CAF signaling pathways thus inhibits tumorigenesis [36].

In the present study we examined the effect of TNFα and IL-17 on glycolysis and growth factor production in colorectal cancer cells. The results indicate that the two cytokines cooperate to increase activity of the glycolytic pathway and to increase production of growth factor(s) that enhance the proliferation/survival of fibroblastic cells.

## Results

**Figure 1:**
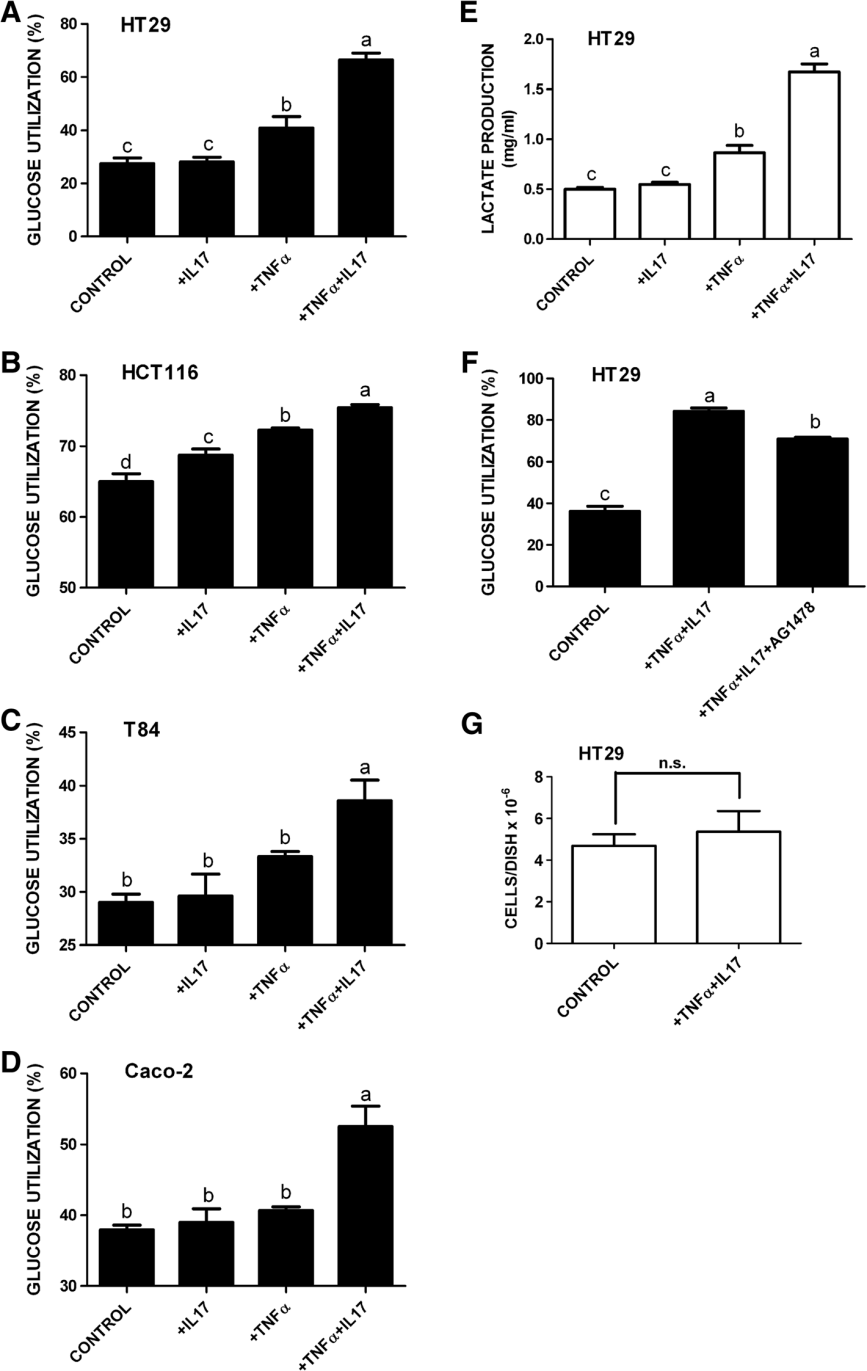
**Effect of TNFα (25 ng/ml) and IL-17 (50 ng/ml) on glucose utilization and lactate production of human colorectal cancer cell lines.** Colorectal cancer cell lines (**A**, HT-29; **B**, HCT116; **C**, T84, and **D**, Caco-2) were treated for 24 h with cytokines as shown. The medium was harvested and assayed for D-glucose. Glucose utilization was expressed as percentage decrease of glucose in the medium. **E**. Production of L-lactate by HT-29 cells. Cells were treated with cytokines as shown. The medium was harvested and assayed for L-lactate. **F**. Effect of EGFR-selective tyrosine protein kinase inhibitor AG1478 on cytokine-stimulated glucose utilization by HT-29 cells. Cells were treated for 24 h with cytokines as shown, with or without AG1478 (1 μM). Culture medium samples were then assayed for D-glucose as in panels A-D. **G**. Effect of TNFα plus IL-17 on HT-29 cell numbers. Cultures were treated with vehicle or cytokines for 24 h, and cells were then harvested and counted. In panels A-F, each bar represents the mean ± SE of three replicate cultures; in panel G each bar represents the mean ± SE of six replicate cultures. Means with different letters were significantly different, *P* < 0.05. In panel G, n.s. = not significant based on *t*-test.

The effect of TNFα, IL-17, and TNFα plus IL-17 on glucose utilization in HT-29 human colorectal cancer cells is shown in Figure 1A. Treatment with TNFα modestly stimulated glucose utilization by the HT-29 cells. Treatment with IL-17 alone had no effect, but IL-17 synergized with TNFα to strongly stimulate glucose utilization. TNFα and IL-17 also cooperatively stimulated glucose utilization by three other human colorectal cancer cell lines, HCT116, T84 and Caco-2 (Figure 1B,C,D). As observed with the HT-29 cells, the effect of TNFα plus IL-17 was synergistic in T84 and Caco-2 cells whereas in HCT116 cells the effect of TNFα plus IL-17 was roughly additive. The effect of TNFα and IL-17 on production of L-lactate by HT-29 cells is shown in Figure 1E. TNFα and IL-17 synergistically stimulated lactate production, indicating that the increased glucose utilization elicited by TNFα plus IL-17 reflected metabolism of glucose through to the end product of the glycolytic pathway, L-lactate.

Treatment of HT-29 cells with TNFα transactivates the EGF receptor (EGFR), and this effect is augmented by IL-17 [30,37]. Therefore, it was possible that the effect of the two cytokines on glucose metabolism might be mediated by EGFR signaling. In support of this notion, EGF has been shown to simulate glucose metabolism in other cells [38,39]. The EGFR-selective receptor tyrosine kinase inhibitor AG1478 (1 μM), which completely inhibits EGFR signaling at this high concentration [40], only modestly inhibited the stimulation of glucose metabolism by TNFα plus IL-17 (Figure 1F). Thus signaling via pathway(s) other than the EGFR pathway appears to be involved in the regulation of glucose metabolism. Treatment of HT-29 cells for 24 h with TNFα plus IL-17 did not significantly affect cell numbers (Figure 1G).

**Figure 2:**
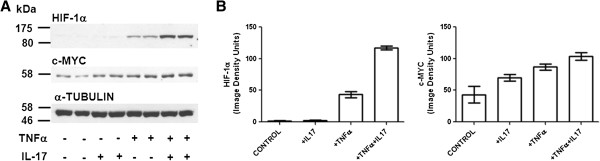
**Effect of TNFα and IL-17 on HIF-1α and c-myc expression in HT-29 cells.** Duplicate serum-free cultures of HT-29 cells were treated for 4 h with TNFα (25 ng/ml) and/or IL-17 (50 ng/ml), or vehicle (control cultures), as indicated. Protein extract samples (60 μg of cellular protein/lane) were run in 7.5% gels. (**A**). Western blots were prepared and probed for HIF-1α, c-myc, or α-tubulin. (**B**). Results were quantified by image analysis. Each bar represents the mean of results obtained for duplicate cultures; the error brackets indicate the range for the duplicates.

Stimulation of glycolysis in cancer cells under hypoxic conditions is thought to be mediated largely by activation of the transcription factor HIF-1α, a master regulator of genes encoding a number of components of the glycolytic pathway [11,12]. The transcription factor c-myc also positively regulates several of these genes [13-15]. The effect of a 4 h treatment with TNFα, IL-17, or TNFα + IL-17 on expression of HIF-1α and c-myc protein is shown in Figure 2. IL-17 synergized with TNFα to increase expression of HIF-1α and also cooperated with TNFα to increase the expression of c-myc (Figure 2).

**Figure 3:**
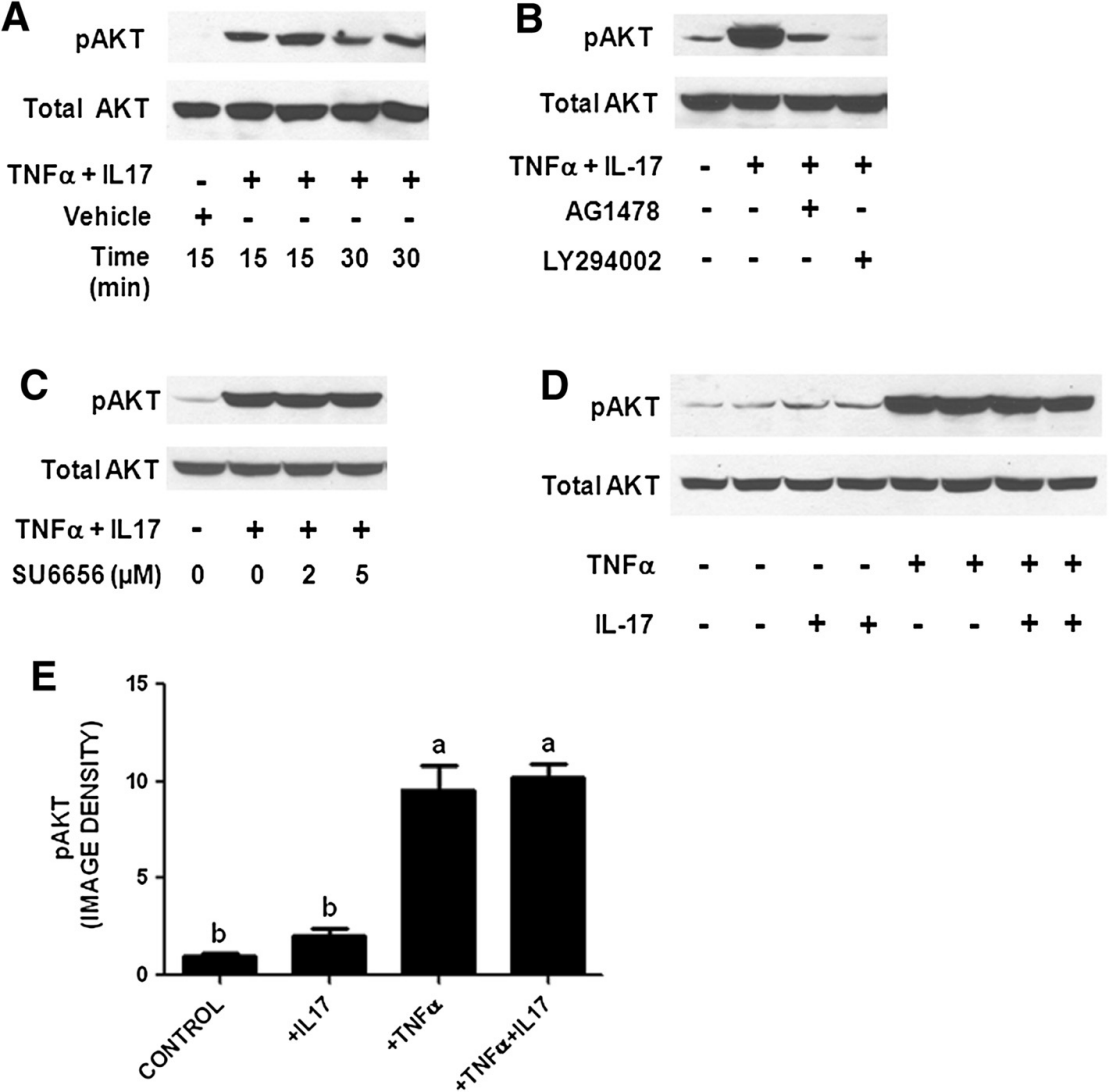
**Effect of TNFα and IL-17 on AKT phosphorylation on Ser473.** Cultures of HT-29 cells were incubated in serum-free medium for 24 h and then treated with vehicle, TNFα (25 ng/ml), IL-17 (50 ng/ml), or TNFα + IL-17. Western blots were prepared and probed with rabbit monoclonal antibody specific for phospho-AKT (Ser473) or whole AKT. **A**. Time course for AKT phosphorylation. In subsequent experiments (**B** - **E**) cells were treated for 15 min. **B**. Effect of EGFR inhibitor AG1478 (1 μM) or PI3K inhibitor LY294002 (20 μM) on AKT phosphorylation. **C**. Effect of Src inhibitor SU6656 on AKT phosphorylation. **D**. Effect of vehicle, TNFα, IL-17, or TNFα + IL-17 on AKT phosphorylation. **E**. Effect of vehicle, TNFα, IL-17, or TNFα + IL-17 on AKT phosphorylation, quantified by image analysis. Each bar represents the mean ± SE of four replicate cultures. Means with different letters were significantly different, *P* < 0.05.

The PI3K-AKT signaling pathway has been reported to play a major role in mediating the regulation of HIF-1α expression in cancer [13] and in response to growth factors [16,17]. We therefore considered the possibility that PI3K-AKT signaling might mediate the effect of TNFα + IL-17 on HIF-1α expression. TNFα + IL-17 dramatically increased the phosphorylation of AKT in HT-29 cells, with a maximal effect observed at 15 min (Figure 3A). This effect was entirely blocked by the PI3K inhibitor LY294002 and substantially inhibited by the EGFR inhibitor AG1478 (Figure 3B), but was unaffected by the Src inhibitor SU6656 Figure 3C. This result suggested that transactivation of EGFR contributed to PI3K pathway activation in response to TNFα + IL-17, but that Src pathway signaling, which sometimes mediates EGFR transactivation [41], was not involved. TNFα strongly increased AKT phosphorylation (Figure 3D,E). In contrast, IL-17 had a very modest effect that did not attain statistical significance, and it also did not significantly augment TNFα-stimulated AKT phosphorylation (Figure 3D,E). Therefore, activation of the PI3K signaling pathway might contribute to TNFα-mediated stimulation of HIF-1α expression, but it did not account for the cooperative effect of IL-17 in combination with TNFα shown in Figure 2.

**Figure 4:**
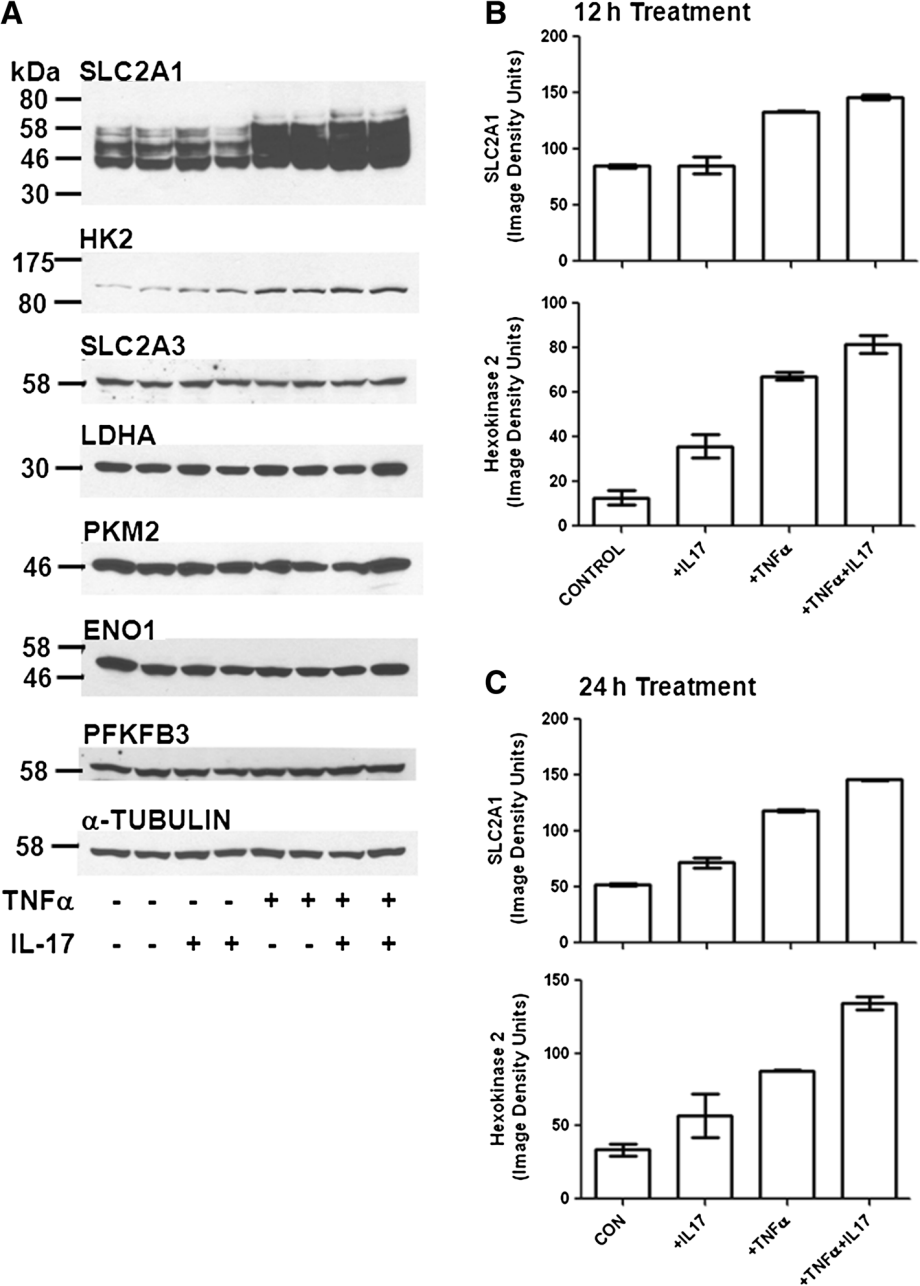
**Effect of TNFα and IL-17 on expression of glycolytic pathway components.** Duplicate serum-free cultures of HT-29 cells were treated for 12 h or 24 h with TNFα (25 ng/ml) and/or IL-17 (50 ng/ml), or vehicle (control cultures), as indicated. Protein extract samples (60 μg of cellular protein/lane) were run in 7.5% gels. (**A**). Western blots were prepared with extracts of cells treated for 12 h and probed for SLC2A1, HK2, SLC2A3, LDHA, PKM2, ENO1, PFKFB3, and α-tubulin. (**B**). Results for SLC2A1 and HK2 were quantified by image analysis. Each bar represents the mean of results obtained for duplicate cultures; the error brackets indicate the range for the duplicates. (**C**). Western blots were prepared for HT-29 cells treated for 24 h, and the results for SLC2A1 and HK2 were quantified by image analysis. Each bar represents the mean of results obtained for duplicate cultures; the error brackets indicate the range for the duplicates.

The results shown in Figure 2 suggested that the effect of TNFα and IL-17 on glycolysis might be mediated by increased expression/activation of HIF-1α and c-myc, leading to transcriptional induction of genes encoding components of the glycolytic pathway. To test this idea, we examined the effect of TNFα and IL-17 on expression of six components of the pathway: the glucose transporters SLC2A1 (Glut1) and SLC2A3 (Glut3), hexokinase-2 (HK2), enolase-1 (ENO1), pyruvate kinase M2 (PKM2), and lactate dehydrogenase A (LDHA). These correspond to the first two steps of the glycolytic pathway (glucose transport and HK2), and the last three steps (ENO1, PKM2, and LDHA). We also examined the effect of TNFα on expression of 6-phoshofructo-2-kinase/fructose-2,6-bisphosphatase-3 (PFKFB3), which produces fructose-2,6-bisphosphate, a major allosteric regulator of the glycolytic pathway enzyme 6-phosphofructo-1-kinase [42]. The genes encoding all seven proteins are targets of transcriptional activation by HIF-1α [12,42-44], and three of them, (SLC2A1, HK2, and LDHA) are also well documented targets of c-myc [13-15]. The results (Figure 4A,B) indicated that in cells treated for 12 h, TNFα and IL-17 cooperatively increased the expression of SLC2A1 and HK2, but did not regulate the expression of SLC2A3, ENO1, PKM2, LDHA, or PFKFB3. In the case of SLC2A1, IL-17 alone had no effect, but it gave a small but reproducible increase in the induction by TNFα. In the case of HK2, IL17 and TNFα both increased expression, and the two cytokines together had a greater effect than either alone (Figure 4A,B). Very similar results were obtained with cells treated for 24 h (Figure 4C).

**Figure 5:**
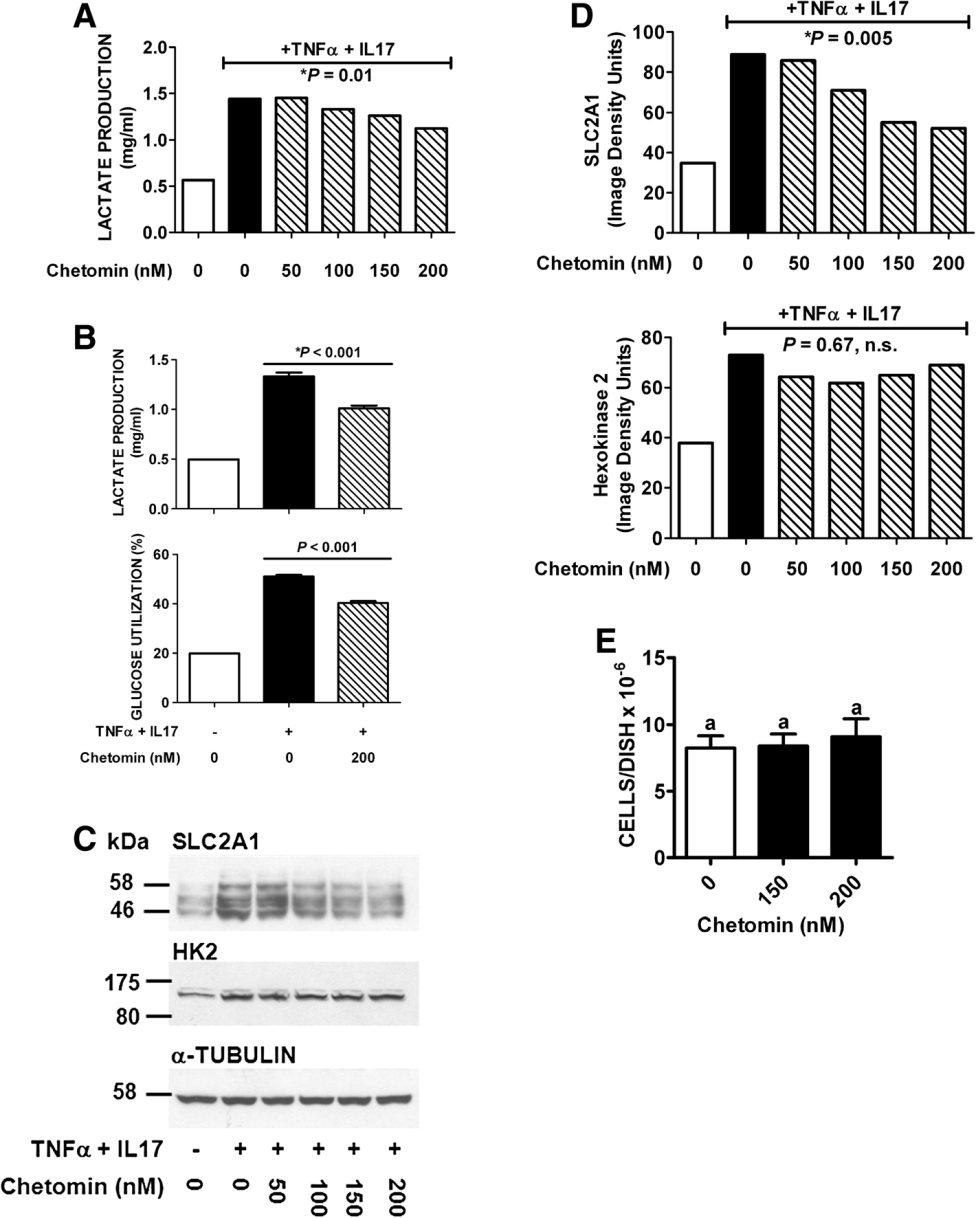
**Inhibition by chetomin of cytokine-stimulated lactate production, glucose utilization, and expression of SLC2A1 and HK2 by HT-29 cells.** Serum-free cultures of HT-29 cells were pre-treated with chetomin or vehicle for 4 h, and TNFα (25 ng/ml) + IL-17 (50 ng/ml) was then added to some of the cultures for an additional 12 h. Medium samples were then collected for lactate and glucose assays, and protein extract samples of cell monolayers (30 μg of cellular protein/lane) were run in 7.5% gels. (**A**). Effect of increasing concentrations of chetomin on lactate production. Analysis of the data by linear regression showed a significant dose-dependent inhibition of lactate production by chetomin, *P* = 0.01. (**B**). Inhibition of lactate production (top) and glucose utilization (bottom) by 200 nM chetomin. Solid black and cross-hatched bars represent the means ± SE of assays performed with medium samples from quadruplicate cultures. Chetomin significantly inhibited lactate production and glucose utilization in cells stimulated by TNFα + IL-17, *P* < 0.001. (**C**). Western blots of cell extract proteins were probed for SLC2A1, HK2, or α-tubulin. (**D**). Results of Western blots shown in panel C were quantified by image analysis. Analysis of the data by linear regression showed significant dose-dependent inhibition by chetomin of SLC2A1 (*P* = 0.005) but not HK2 (*P* = 0.67, n.s.) expression. (**E**). Effect of chetomin on HT-29 cell numbers. Cultures were treated with vehicle or chetomin for 16 h, and cells were then harvested and counted. Each bar represents the mean ± SD of results obtained with six cultures. Bars with different letters were significantly different, *P* < 0.05.

Chetomin is a low molecular weight compound that inhibits transcriptional activation by HIF-1α by blocking its binding of the co-activator p300 [45]. The effect of chetomin on cytokine-induced lactate production and expression of SLC2A1 and HK2 is shown in Figure 5. Chetomin partially inhibited the stimulation of lactate production by TNFα + IL-17 in a dose-dependent manner (Figure 5A). In another experiment performed in quadruplicate, 200 nM chetomin inhibited lactate production by 24% in cells stimulated with TNFα + IL-17, and a commensurate inhibition of glucose utilization was also observed (Figure 5B). A partial inhibition by chetomin of cytokine-stimulated SLC2A1 expression was also observed (Figure 5C,D). The effective chetomin dose range of 50-200 nM was very similar to that required for inhibition of HIF-1α action in other human cancer cell lines [46]. In contrast chetomin had little or no effect on HK2 expression (Figure 5C,D). Finally, chetomin had no significant effect on cell numbers under the conditions of our experiments (Figure 5E).

**Figure 6:**
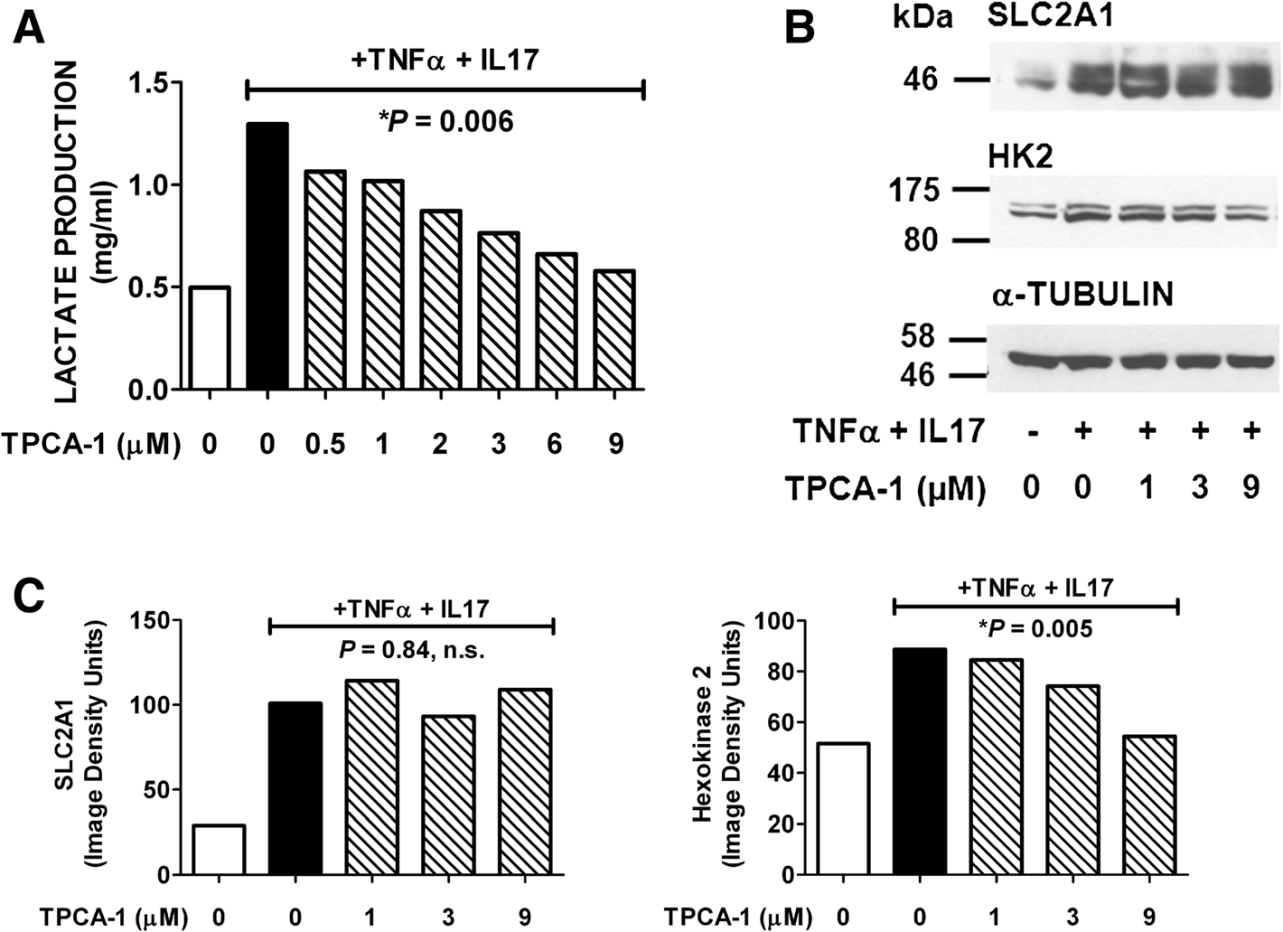
**Inhibition by TPCA-1 of cytokine-stimulated lactate production and expression of SLC2A1 and HK2 by HT-29 cells.** Serum-free cultures of HT-29 cells were treated for 12 h with TNFα (25 ng/ml) + IL-17 (50 ng/ml), in the presence of TPCA-1 or vehicle, as shown. Medium samples were then collected for lactate assays, and protein extract samples of cell monolayers (30 μg of cellular protein/lane) were run in 7.5% gels. (**A**). Effect of increasing concentrations of TPCA-1 on lactate production. Analysis of the data by linear regression showed a significant dose-dependent inhibition of lactate production by TPCA-1, *P* = 0.006. (**B**). Western blots of cell extract proteins were probed for SLC2A1, HK2, or α-tubulin. (**C**). Results of Western blots shown in panel B were quantified by image analysis. Analysis of the data by linear regression showed significant dose-dependent inhibition by TPCA-1 of HK-2 (*P* = 0.005) but not SLC2A1 (*P* = 0.84, n.s.) expression.

NF-κB plays a major role in mediating transcriptional induction of genes encoding several chemokines in cells treated with TNFα + IL-17 [30,31]. Several studies have documented that TNFα activates NF-κB in HT-29 and other human colorectal cancer cell lines [30,47,48]. The protein kinase IKKβ is a key component of the pathway for NF-κB activation by TNFα [49]. To investigate the possibility that NF-κB might participate in the induction of SLC2A1 and/or HK2, the effect of the IKKβ-selective inhibitor TPCA-1 [50] on cytokine-induced lactate production and expression of SLC2A1 and HK2 was tested. The results indicated that TPCA-1 strongly inhibited the cytokine-stimulated component of lactate production by HT-29 cells (Figure 6A). A similar strong inhibition of cytokine-stimulated HK2 expression was also observed (Figure 6B,C). In contrast TPCA-1 had little or no effect on SLC2A1 expression (Figure 6B,C). The combined results presented in Figures 5 and 6 suggest that HIF-1α plays a role in the induction of SLC2A1 by TNFα + IL-17, and that NF-κB plays a role in the induction of HK2.

**Figure 7:**
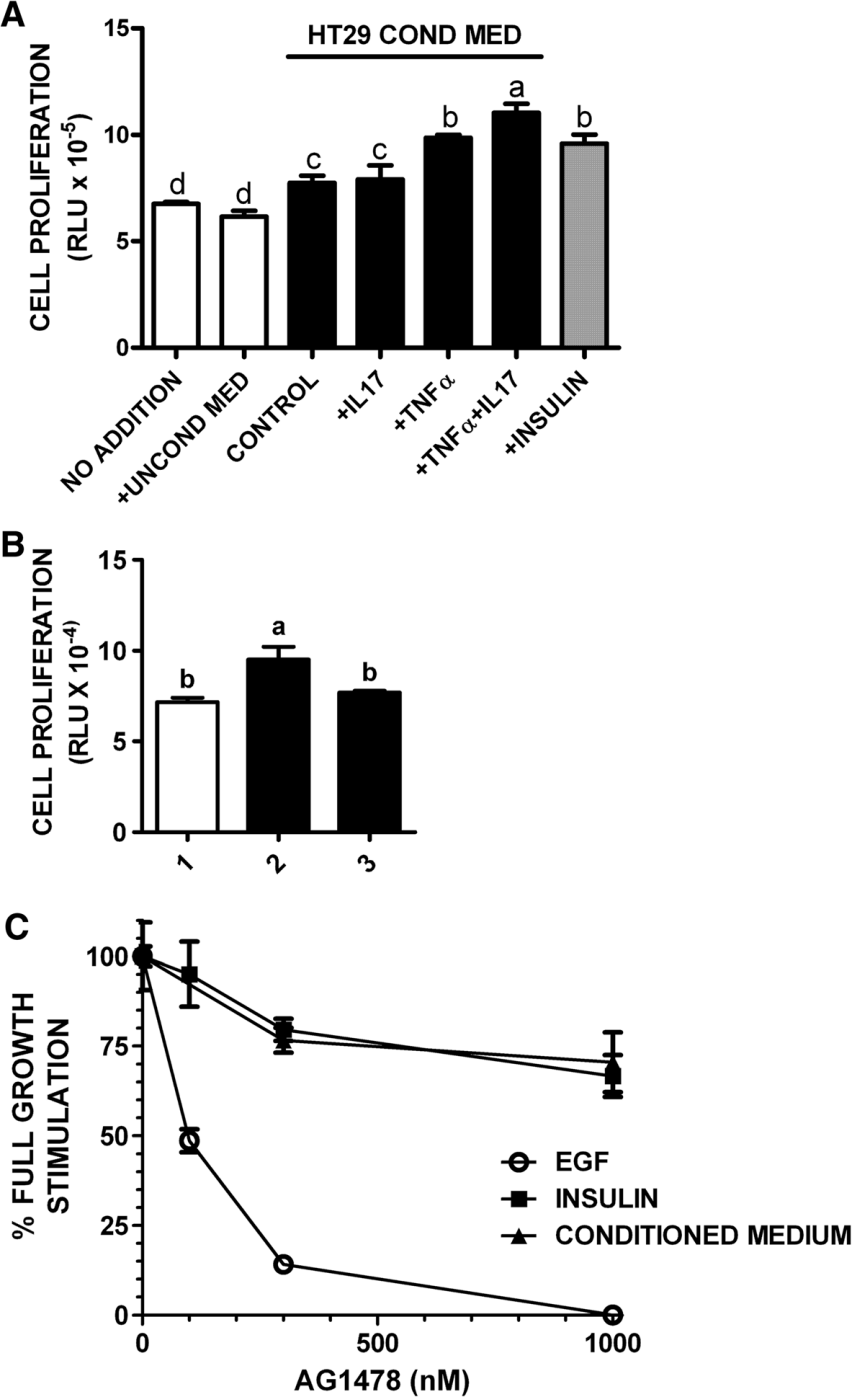
**Medium conditioned by HT-29 cells treated with TNFα and IL-17 stimulates growth/survival of NIL8 cells.** (**A**). NIL8 cells were treated for 24 h with nothing, or 10 μl unconditioned medium (open bars), or insulin (1 μg/ml) (filled gray bar), or 10 μl of medium conditioned by cultures of HT-29 cells treated with vehicle (control), TNFα, IL-17, or TNFα + IL-17 (filled black bars). Cell growth/survival of the NIL8 cells was then assayed (Methods). Each bar is the mean of quadruplicate assays ± SE. Means with different letters were significantly different, *P* < 0.05. (**B**). Cell growth/survival of NIL8 cells treated with: Bar 1: unconditioned McCoys 5A medium; Bar 2: medium conditioned by HT-29 cells stimulated with 25 ng/ml TNFα + 50 ng/ml IL-17 for 24 h; Bar 3: unconditioned medium containing 25 ng/ml TNFα + 50 ng/ml IL-17. Means with different letters were significantly different, *P* < 0.05. (**C**). NIL8 cells were treated for 24 h with EGF (300 ng/ml) (○), insulin (10 μg/ml) (■), or 10 μl of medium conditioned by HT-29 cells stimulated with TNFα + IL-17 (▲), plus increasing concentrations of AG1478. Cell growth/survival vs. unstimulated control NIL8 cells was then assayed (Methods). Data were plotted as % growth stimulation observed in the absence of AG1478. Results show the means of quadruplicate assays ± SE.

Paracrine signaling between tumor cells and tumor-associated stromal cells plays an important role in carcinogenesis. We next used the NIL8 fibroblastic cell line to determine whether TNFα and IL-17 regulated production of growth factors that are active in fibroblastic cells. The NIL8 cells have been shown previously to respond to a variety of growth factors that stimulate the proliferation of fibroblasts [51-53]. The results (Figure 7A) demonstrated that TNFα stimulated growth factor release by HT-29 cells. IL-17 alone had no effect but enhanced the effect of TNFα on growth factor production. Insulin was used as a positive control in the NIL8 bioassay (Figure 7A). TNFα and IL-17 at the maximum possible concentration remaining in the conditioned medium had no significant effect on growth/survival of the NIL8 cells, showing that the growth factor activity was not attributable to residual TNFα plus IL-17 (Figure 7B).

As noted above, TNFα transactivates the EGF receptor (EGFR) in HT-29 cells, and this effect is augmented by IL-17 [30,37]. The TNFα-elicited component of this effect is reported to involve release of the EGFR ligand transforming growth factor-α (TGF-α) and its subsequent activation of EGFR [37]. Thus, it was possible that the growth factor activity detected in the NIL8 bioassay corresponded to EGFR ligand(s) released by the HT-29 cells in response to TNFα plus IL-17. To determine whether this was the case, we examined the effect of the selective EGFR tyrosine kinase inhibitor AG1478 on activity of the HT-29-derived growth factor in NIL8 cells (Figure 7C). As controls we used EGF, which acts entirely via EGFR, and insulin, which at high concentrations acts via the insulin receptor (IR) and IGF receptor-1 (IGFR1) [54]. As expected, AG1478 strongly inhibited EGF action in the NIL8 cells, with 50% inhibition observed at 100 nM AG1478 (Figure 7C). In contrast, AG1478 at higher concentrations weakly inhibited insulin signaling, presumably due to non-specific inhibition of IR and IGFR1 tyrosine kinase activity. The AG1478 inhibition curve for the activity produced by HT-29 cells was identical to that of insulin, indicating that this growth factor does not act primarily through EGFR (Figure 7C).

## Discussion

Chronic inflammation is a well-known risk factor for colorectal cancer, but molecular mechanisms underlying the effects of inflammation on carcinogenesis are incompletely understood. Procarcinogenic effects of cytokines produced by inflammatory cells are thus of considerable interest. TNFα and IL-17 are commonly found together in the context of both acute and chronic inflammation; therefore, the effects of TNFα + IL-17 are biologically relevant to the inflammation-cancer interface. The present results show that TNFα and IL-17 synergistically stimulate glycolysis and growth factor production by human colorectal cancer cells, effects that could contribute to the positive effect of inflammation on carcinogenesis.

The transcription factor HIF-1α is a master regulator of genes encoding components of the glycolytic pathway, and c-myc also positively regulates some of these genes. TNFα cooperatively induced the expression of both HIF-1α and c-myc in HT-29 cells. Of particular interest, the effect of TNFα and IL-17 on HIF-1α was synergistic and therefore resembled the synergistic effect of the two cytokines on glycolysis in HT-29, T84 and Caco-2 cells (Figure 1A,C,D,E). We initially hypothesized, therefore, that HIF-1α produced in response to TNFα plus IL-17 might globally induce transcription of genes encoding components of the glycolytic pathway, and that c-myc might also contribute to this induction. To test this idea the effects of TNFα and IL-17 on expression of six components and one regulator of the glycolytic pathway were examined. The genes encoding all seven proteins are known targets of HIF-1α [12,41-43], and three of them (SLC2A1, HK2 and LDHA) are also well-documented targets of c-myc [13-15]. Surprisingly, TNFα and IL-17 selectively induced expression of SLC2A1 and HK2 but did not regulate the expression of SLC2A3, ENO1, PKM2, LDHA, or PFKFB3. The glucose transporter SLC2A1 (also known as Glut1) facilitates the uptake of glucose, and HK2 catalyzes the first step in glycolysis, phosphorylation of D-glucose to yield D-glucose-6-phosphate.

HIF-1α was initially identified as a mediator of effects of hypoxia, and these effects vary in a cell type-specific manner. Relevant to the present results, selective induction of HK2 and pyruvate dehydrogenase kinase-1 (PDK1) by hypoxia (and HIF-1α and c-myc) was observed in a previous study of human P493-6 B-lymphoblastoid cells, a model for human Burkitt’s lymphoma [55]. In these cells, several other putative HIF-1α/c-myc targets including ENO1 and LDHA were not regulated by hypoxia, HIF-1α or c-myc [55]. Similarly, in MCF7 breast cancer cells SLC2A1, HK2, and PFKFB3 but not ENO1 or LDHA were strongly induced by hypoxia [56]. In contrast, hypoxia and HIF-1α induced expression of ENO1 and LDHA in another human cancer cell line, HEP3B hepatoma cells [57,58]. The difference in response of various HIF-1α target genes to changes in HIF-1α observed in different cancer cell lines remains to be investigated further. Possible explanations include higher or lower affinities of different binding sites for HIF-1α, sequence context or chromatin configuration of the binding sites, different basal levels of expression of HIF-1α target genes in different cancer cells, or differences in experimental protocols from study to study.

The PI3K-AKT signaling pathway plays an important role in regulating HIF-1α expression in cancer [13] and in response to growth factors [16,17]. In the present study AKT was activated in response to TNFα but not IL-17. This activation of AKT was largely mediated by EGFR transactivation, as it was strongly inhibited by the selective EGFR tyrosine kinase inhibitor AG1478. However, AG1478 had a minimal effect on stimulation of glycolysis by TNFα + IL-17 (Figure 1F), that resembled the ~25% non-specific inhibition by 1 μM AG1478 of receptor tyrosine kinases other than EGFR (Figure 7C). This indicates that signaling events other than EGFR transactivation play an important role in induction of glycolysis by TNFα + IL-17.

Chetomin is a low molecular weight compound produced by the mold Chaetomium cochliodes that inhibits transcriptional activation by HIF-1α [45]. Chetomin elicited a dose-dependent partial inhibition of TNFα plus IL-17-stimulated lactate production, which correlated with inhibition of SLC2A1 but not HK2 induction. The effective chetomin dose range and degree of inhibition of cytokine-stimulated SLC2A1 expression was remarkably similar to that seen for inhibition by chetomin of hypoxia-stimulated expression of two other HIF-1α targets, CA9 and VEGF, in HT1080 human fibrosarcoma cells [46]. The results presented here therefore suggest that HIF-1α-mediated induction of SLC2A1 plays a role in the stimulation of glycolysis by TNFα + IL-17. The SLC2A1 gene has been shown previously to be a HIF-1α target , and ChIP-Seq and ChIP-Chip analysis has identified a HIF-1α binding site upstream of the SLC2A1 gene [59-61].

IL-17 synergizes with TNFα to induce expression of chemokines such as CXCL1, CXCL8, and CCL20 in HT-29 and other cells [30]. Major mechanisms for this induction are activation of the canonical NF-κB signaling pathway by TNFα, resulting in increased chemokine gene transcription, and IL-17-mediated stabilization of chemokine mRNAs [31,32]. The possibility was therefore considered that NF-κB might play a role in the induction of glycolysis by TNFα + IL-17. The protein kinase IKKβ is a key component of the canonical NF-κB signaling pathway, and TPCA-1 is a selective inhibitor of IKKβ [50]. Interestingly, TPCA-1 strongly inhibited TNFα plus IL-17-stimulated lactate production and induction of HK2 but not SLC2A1. These results suggest that NF-κB-mediated induction of HK2 plays a role in the stimulation of glycolysis by TNFα + IL-17. The ENCODE project has accomplished human genome-wide mapping of binding sites for NF-κB and a number of other transcription factors [62]. The results of the ENCODE project are now displayed in the human genome database (http://genome.ucsc.edu). Within intron 1 of the HK2 gene, there is a sequence that is bound by NF-κB in four human lymphoblastoid cell lines and that maps to a DNase-1 hypersensitive site. In the current assembly (hg19) of the human genome sequence, the coordinates for this sequence are chr 2: 75076257-75076681. This interval contains a sequence on the opposite strand 5′-GGGGCATTCC-3′, which agrees with the consensus NF-κB binding sequence GGGRNNYYCC [63]. It is possible that this site might play a role in induction of HK2 expression by TNFα + IL-17.

Evidence indicates that cancer-associated fibroblasts (CAF) and tumor cells form a reciprocal positive feedback loop in which tumor cells produce factors that promote activation, proliferation, and chemotaxis of CAF, which in turn produce factors that enhance tumor cell proliferation [33-36]. The results presented here demonstrate that IL-17 synergizes with TNFα to stimulate the production by HT-29 cells of a factor that increases proliferation/survival of fibroblastic cells. Similar to the effect of TNFα and IL-17 on glycolysis, IL-17 alone did not affect production of the factor but it significantly enhanced TNFα-stimulated factor production. This factor does not appear to act primarily via EGFR, and its identity remains to be investigated.

Human colorectal tumors contain CD11c^+^ macrophage-like cells that produce TNFα [29], and Th17 cells that produce IL-17 [26,27]; thus, the two cytokines are both expressed in colorectal tumors. Moreover, increased TNFα expression is correlated with increased diameter and extent of primary tumors [29], and studies of mouse models have indicated that IL-23/IL-17 signaling plays a key role in colorectal tumor development [28]. In the present study we investigated mechanisms for the apparent pro-carcinogenic effect of TNFα and IL-17.

## Conclusions

Based on the results presented here, stimulation of glycolysis and growth factor production are two mechanisms by which TNFα and IL-17 might cooperate in promoting colorectal carcinogenesis.

## Methods

### Materials

Human recombinant TNFα (210-TA) and IL-17A (317-IL) were from R&D Systems. Anti-PKM2 mouse monoclonal antibody (5D2-3B3) and anti-SLC2A1 and SLC2A3 polyclonal antibodies used for Western blotting were from Millipore. All other primary antibodies used for Western blotting were from Cell Signaling Technology. HRP-conjugated secondary antibodies for Western blotting were from Vector Laboratories. SuperSignal West Pico Chemiluminescent Substrate, obtained from Thermo Scientific, was used for detection of HRP-secondary antibodies in Western blots. Chetomin, TPCA-1, and AG1478 were from Sigma-Aldrich. The glucose (HK) detection reagent was from Sigma-Aldrich (#G3293). Rabbit muscle lactate dehydrogenase (25 kU/mg) used for assaying L-lactate was from EMD Millipore.

### Cell culture

Human colorectal cancer (CRC) cell lines HT-29, T84, HCT119, and Caco-2 were cultured at 37 C in McCoy’s 5A medium supplemented with 15 mM HEPES, 10% FBS, penicillin (100 U/ml) and streptomycin (100 μg/ml). The NIL8 hamster fibroblastic cell line [51-53] was cultured in MEM supplemented with 10% FBS, non-essential amino acids, penicillin (100 U/ml) and streptomycin (100 μg/ml). Cell counts were performed with a hemocytometer.

### Glucose utilization, lactate production, and growth factor experiments

CRC cells were plated at a density of 2 × 10^6^ cells per 3.5 cm diameter well (6 well plates) in McCoy’s 5A medium with 10% FBS and antibiotics. After 48 hours the medium was changed to serum-free McCoys 5A with antibiotics, and cells were cultured for an additional 24 h. At this time the cells had grown to confluent monolayers. The medium was aspirated, and the cultures were washed once with PBS. Fresh serum-free McCoys 5A medium plus antibiotics was then added to each well. TNFα (25 ng/ml), IL-17 (50 ng/ml), or vehicle was then added as indicated, and the cells were cultured for an additional 24 h. The medium was then harvested and stored at -80 C prior to being assayed for glucose, L-lactate, or growth factor activity.

### Glucose and L-lactate assays

For assaying the concentration of D-glucose in culture media, the glucose (HK) assay reagent (Sigma-Aldrich #G3293) was utilized. This method uses two enzymes, hexokinase and glucose-6-phosphate dehydrogenase. Hexokinase first catalyzes conversion of D-glucose to glucose-6-phosphate. Glucose-6-phosphate dehydrogenase then catalyzes the reaction of glucose-6-phosphate and NAD^+^ to produce 6-phosphogluconate and NADH. Progress of the reaction was monitored spectrophotometrically by measuring production of NADH, which absorbs light at 340 nm. The concentration of glucose was determined using a standard curve. The concentration of D-glucose in McCoy’s 5A medium is 3 mg/ml. Glucose utilized by the cells during the 24 h incubation period was calculated as the difference between the starting glucose concentration (i.e. in medium not incubated with cells) and the final glucose concentration in medium incubated with cells, and was expressed as a percentage of the starting concentration.

The production of L-lactate by cultured cells was assayed enzymatically with rabbit muscle lactate dehydrogenase (EMD Millipore Corp, 25 kU/mg), as described in ref. [64]. The starting medium, serum-free McCoy’s 5A, does not contain L-lactate. The concentration of L-lactate in medium after cell culture was determined using a standard curve.

### Growth factor assays

Bioassays of growth factor activity in cell culture media were performed using the NIL8 hamster fibroblastic cell line, which responds to a variety of growth factors that are active in fibroblasts [51-53]. The Lonza ViaLight Plus cell proliferation kit was used for these assays. The kit measures the amount of ATP in monolayer cultures and therefore measures cell proliferation/survival. Briefly, NIL8 cells were plated in 96 well cell culture plates in MEM plus 0.3% serum, non-essential amino acids and antibiotics. After 24 h, additions of cell culture medium samples were made as indicated. The cells were then incubated for 24 h, lysed, and the amount of ATP per well was determined using the manufacturer’s protocol. Insulin, which stimulates DNA synthesis in NIL8 cells [51-53], was used as a positive control.

### Protein expression experiments and AKT phosphorylation assays

The effect of cytokines on expression of specific cellular proteins and on AKT phosphorylation was determined by Western blot analysis of whole cell extracts by standard methods as described previously [65]. Cells were plated in 6 cm dishes at a density of 5-7 × 10^6^ cells/dish, and the cultures were incubated at 37 C until confluent. The cultures were washed with PBS, fresh serum-free medium was added, and the cultures were incubated for 24 h at 37 C. TNFα (25 ng/ml) and/or IL-17 (50 ng/ml), or vehicle (control cultures), was then added, and incubation was continued for the time indicated. The NETN extraction buffer [65] used for preparing cell extracts was supplemented with a protease inhibitor mix obtained from Sigma-Aldrich (#P8340) and phosphatase inhibitors sodium fluoride, disodium β-glycerophosphate, sodium pyrophosphate, and sodium vanadate. In all experiments the concentration of protein in each cell extract was determined by the method of Lowry [66], and 30 or 60 μg of extract protein was loaded in each lane of the gel. For Western blot analysis of SLC2A1 expression, samples were not boiled prior to loading the gels, to prevent aggregation of SLC2A1 protein [67]. For measurements of AKT phosphorylation, Western blots were probed with monoclonal antibodies that specifically recognize phospho-AKT (Ser473) or total AKT (Cell Signaling #4060S and #4691S). Methods for blotting the gels and probing the blots were as described previously [65].

### Statistics

The unpaired *t*-test was used for comparison of two means. For comparison of more than two means, data were subjected to one-way ANOVA followed by the Student-Newman-Keuls multiple comparison test. Linear regression analysis was performed for evaluation of inhibitor data, with *P* < 0.05 used as a cut-off for significance of a downward trend in assay result plotted as a function of increasing inhibitor concentration.

## Abbreviations

TNFα: Tumor necrosis factor α; IL-17: Interleukin 17; HIF-1α: Hypoxia inducible factor 1α; NF-κB: Nuclear factor κB; HK2: Hexokinase-2; ENO1: Enolase-1; PKM2: Pyruvate kinase M2; LDHA: Lactate dehydrogenase A; PFKFB3: 6-phoshofructo-2-kinase/fructose-2,6-bisphosphatase isoform 3; PI3K: Phosphatidylinositol 3-kinase; EGFR: Epidermal growth factor receptor; CAF: Stromal cancer-associated fibroblasts.

## Competing interests

The authors declare that they have no competing interests.
